# The causal impact of gut microbiota on allergic rhinitis mediated by cerebrospinal fluid metabolites: A study based on Mendelian randomization and mediation analysis

**DOI:** 10.1097/MD.0000000000049384

**Published:** 2026-07-03

**Authors:** Guoteng Zhao, Peizheng Yan, Ran Qiu, Mingzhe Zhao, Zhihao Zhang, Zhenguo Wang

**Affiliations:** a Institute of Traditional Chinese Medicine Literature and Culture, Shandong University of Traditional Chinese Medicine, Jinan, China; b Key Laboratory of Traditional Chinese Medicine Classical Theory, Ministry of Education, Shandong University of Traditional Chinese Medicine, Jinan, China; c School of Pharmacy, Shandong University of Traditional Chinese Medicine, Jinan, China.

**Keywords:** allergic rhinitis, brain functional networks, cerebrospinal fluid metabolites, gut microbiotas, gut-brain-immune axis, Mendelian randomization

## Abstract

In recent years, the role of gut microbiota (GM) in the pathogenesis of allergic diseases (ADs) has garnered increasing attention, yet the influence of the brain and its metabolites (such as cerebrospinal fluid [CSF] metabolites) remains unclear. This study aims to investigate whether GM mediates its effect on allergic rhinitis (AR) through CSF metabolites and to quantify this mediating effect, while systematically evaluating the causal relationships between brain functional networks/CSF metabolites and multiple ADs to elucidate the potential roles of brain-related factors in allergic mechanisms. Using large-scale genome-wide association study data, including GM (n = 7738), brain functional networks (n = 47,276), CSF metabolites (n = 291), and 7 categories of AD (Finnish R12 database), we applied Mendelian randomization (MR) for causal inference. Specifically, we performed: mediation MR to test the GM–CSF metabolites–AR pathway; and 2-sample MR to assess causal relationships between brain functional networks/CSF metabolites and ADs. Genetically predicted analyses revealed significant associations between GM and both CSF metabolites and AR. Mediation MR confirmed that *Peptococcia* abundance exerts a positive causal effect on AR (odds ratio [OR] = 1.55, 95% confidence interval [CI] = 1.23–1.95, *P* = .0002), which was partially mediated by CSF methyl succinoyl-carnitine levels, with a mediation proportion of 9.02%. Extended analyses indicated that CSF metabolites are positively associated with allergic contact dermatitis and pollen allergy: specifically, methyl succinoyl-carnitine levels correlated positively with pollen allergy, and indoleacetate levels with allergic contact dermatitis (both significant after false discovery rate correction). Brain functional network analyses showed allergic urticaria was negatively correlated with 9 functional networks, including the occipital-precuneus and postcentral gyrus. This study is the first, via MR, to demonstrate that GM may partially influence AR risk through CSF metabolites (e.g., methyl succinoyl-carnitine); concurrently, brain functional networks regulate specific ADs. These findings deepen understanding of the “gut-brain-immune” axis mechanism and provide new directions for future precise interventions targeting metabolites and neuroimmune pathways.

## 1. Introduction

Allergic diseases (ADs) are a category of common immune dysregulation disorders involving multiple organs and symptoms. The primary clinical manifestations include itching, erythema, nasal congestion, rhinorrhea, edema, and dyspnea. In severe cases, ADs may lead to critical complications such as shock and asphyxia, posing life-threatening risks. It is reported that over 30% of the global population is affected by 1 or more ADs,^[1]^ resulting in a substantial socioeconomic burden, which is being qualitatively exacerbated by the trend of climate change.^[2]^

The etiology of ADs is complex, with common pathogenic factors including genetic susceptibility and environmental exposures. Factors such as rising temperatures, increased CO_2_ concentrations, non-breastfeeding, or early introduction of certain solid foods can contribute to the development of ADs. The diagnosis and management of allergic rhinitis (AR) remain challenging, as its symptoms often overlap with those of nonallergic rhinitis, and epidemiological data indicate a rising incidence year by year. This necessitates that clinicians integrate detailed medical history and objective testing to optimize diagnostic accuracy.^[3]^

Genetic studies suggest that the onset of ADs is frequently associated with specific genetic variations, which influence disease progression, severity, and individual responses to drug therapies.^[4]^ Although the pathogenesis of ADs, particularly AR, has been extensively studied, a complete understanding remains elusive. Current research predominantly focuses on the roles of T-helper type 1and T-helper type 2 cells, often overlooking other critical immune cells such as group 2 innate lymphoid cells, B cells, T cells, and macrophages. These cells participate in inflammatory responses through cytokine production, yet their specific mechanisms require further investigation.^[5]^

In recent years, neuroimmune communication mechanisms (e.g., neuropeptide-mediated inflammation) have gained increasing attention. The specific interactive networks involved in AR hold considerable potential for exploration, though research in this area remains relatively limited.^[6]^ To date, elucidating the mechanisms underlying AR remains a global challenge. It is reasonable to hypothesize that the inflammatory process in AR may influence the central nervous system (CNS) via the neuro-immune axis, thereby altering the metabolite profile in cerebrospinal fluid.

The majority of microorganisms in the human body colonize the gastrointestinal tract. Gut microbiota (GM) refers to the microbial community inhabiting the gastrointestinal tracts of humans and animals, playing a pivotal role in maintaining host health. GM contributes to preserving intestinal structural integrity and function, modulates the immune system, protects against pathogen invasion, and provides nutritional benefits.^[7]^ Studies have shown that GM communicates bidirectionally with the CNS via the “microbiota-gut-brain axis.” This pathway involves neurotransmission, hormones, the immune system, and other molecular signals, thereby influencing brain function and related disorders.^[8,9]^

Specifically, GM may influence CSF metabolites through multiple pathways. For instance, GM can directly modulate the metabolic environment of the CNS by affecting the blood–brain barrier.^[10]^ In addition, GM may indirectly alter cerebral metabolic processes through immune regulation, leading to changes in the composition of CSF metabolites.^[11,12]^ ADs typically involve immune abnormalities, and GM may exert indirect effects on allergies by regulating immune responses and inflammatory pathways. GM influences the synthesis of neurotransmitters such as 5-hydroxytryptamine, which plays a key role in the gut-brain axis by modulating mood and immune responses. Dysregulation of 5-hydroxytryptamine may be associated with stress-induced immune dysfunction, thereby influencing the development of ADs.^[13,14]^ This study aims to explore the mechanisms by which GM participates in ADs through modulation of the brain and immune system.

CSF metabolites refer to small-molecule metabolic products present in cerebrospinal fluid, originating from metabolic processes in the brain and CNS. They reflect the biochemical state of the nervous system and include various types such as amino acids, lipids, carbohydrates, and nucleotides. These metabolites support cognitive function, synaptic plasticity, and brain structural integrity through core biological pathways, including energy metabolism, neurotransmitter synthesis and degradation, antioxidant defense, and inflammation regulation.^[15,16]^

As key indicators of brain metabolic status, CSF metabolites not only participate in fundamental physiological functions such as cognitive maintenance and neuroprotection but also serve as potential biomarkers and therapeutic targets in various diseases.^[17]^ These functions underscore their importance in neuroscience and clinical medicine, providing a theoretical basis for future interventions targeting metabolic pathways.^[18]^ This “gut-brain-immune” triad suggests that CSF metabolites may act as important mediators through which GM regulates allergic responses, though their causal role has not been systematically validated in human studies.

Simultaneously, neural activity in brain functional areas has been found to engage in bidirectional communication with immune regulation.^[19]^ The regulatory influence of brain functional areas may reside upstream in the causal chain, with effects potentially transmitted through humoral factors rather than acting directly on peripheral allergic responses.^[20]^ Therefore, in subsequent analyses, we performed 2-sample Mendelian randomization (MR) analyses using multiple brain omics data to investigate the causal relationships between brain functional areas and CSF metabolites with ADs.

This study uses a 2-sample MR approach, using genetic variants as instrumental variables, to systematically evaluate the causal effects of GM on ADs, with a particular focus on examining the mediating role of CSF metabolites in this relationship. By quantifying the mediation proportion, we aim to elucidate the potential mechanisms through which GM influences allergies, providing genetic evidence for the development of novel intervention strategies targeting the microbe-metabolism axis.

## 2. Methods

### 2.1. Research design

For the MR analyses, the inverse variance weighting method (IVW) is mainly used to make inferences about the causal effect between exposure and outcome. In the analysis of the article, GM were used as an exposure factor, single nucleotide polymorphisms (SNPs) significantly associated with GM were used as an instrumental variable, AR was used as an outcome variable, and CSF metabolites were included as mediating factors in the analysis. To obtain reliable results, 2-sample MR should fulfill 3 basic assumptions.

1. **Association hypothesis:** the selected SNPs are strongly associated with exposure factors.
2. **Independence assumption:** SNPs must not be associated with potential confounding factors between exposure and outcome.
3. **Exclusivity hypothesis:** SNPs can only affect the outcome through exposure factors.

**Figure 1.**
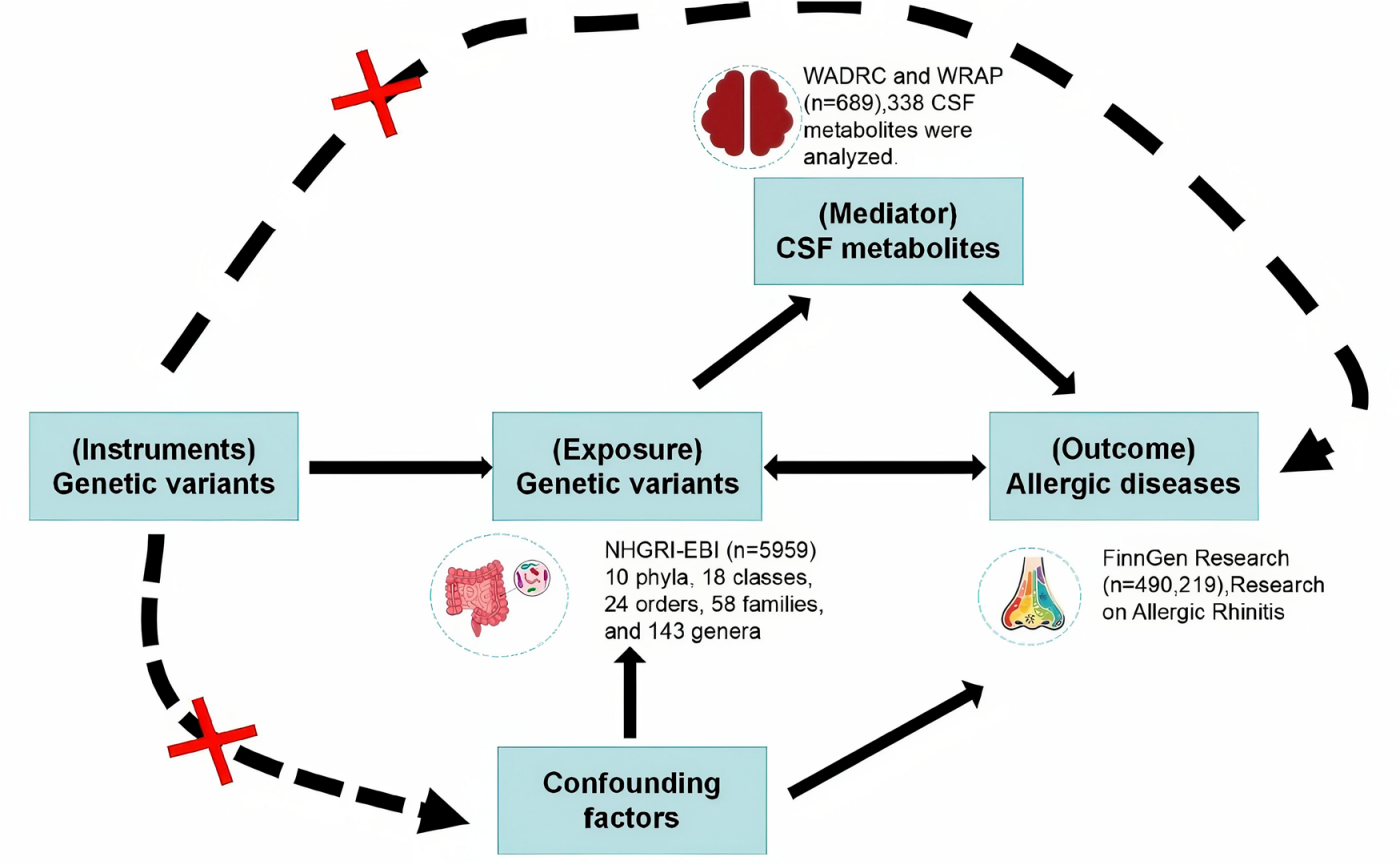
Study design flowchart showing the 2-sample Mendelian randomization (with 3 core assumptions) and mediation analysis, depicting the causal path from gut microbiota (exposure) to allergic diseases (outcome) mediated by cerebrospinal fluid metabolites. CSF = cerebrospinal fluid, NHGRI-EBI = National Human Genome Research Institute–European Bioinformatics Institute (GWAS Catalog), WADRC = Wisconsin Alzheimer’s Disease Research Center, WRAP = Wisconsin Registry for Alzheimer’s Prevention.

Therefore, our study was accomplished using 2-sample, 2-step MR. First, in 2-sample MR, the causal relationship between GM and ADs was analyzed positively and negatively, and the most significant GM without reverse results was screened out. In the first step of the 2-step MR, we used MR to analyze the causal relationship between them (i.e., the most significant GM) and the mediating factors (i.e., CSF metabolites), and screened out the CSF metabolites that were strongly correlated with the disease; in the second step, we conducted a positive MR analysis of the screened CSF metabolites and AR, and finally derived the CSF metabolites that are strongly correlated with the disease, so as to observe the mediating effect of CSF metabolites mediating the influence of GM on ADs. The specific study design is shown in Figure 1.

### 2.2. Data sources

The genome-wide association study (GWAS) data for the GM were obtained from the European Bioinformatics Institute’s GWAS database, accessible at https://www.ebi.ac.uk/gwas/. This database provides detailed records of the characteristics of various GM taxa. A total of 473 taxa were meticulously selected for analysis in this study, covering 10 phyla, 18 classes, 24 orders, 58 families, 143 genera, 213 species, and 7 unclassified microbial groups.

Our research utilized GWAS Catalog datasets focusing on CSF metabolites. The data for the 338 CSF metabolites were obtained from the Wisconsin Alzheimer’s Disease Research Center and Wisconsin Registry for Alzheimer’s Prevention cohort studies. The analysis included both patients and healthy controls, with a total of 689 participants initially involved. The metabolites were identified and quantified using nontargeted metabolomics with ultra performance liquid chromatography-tandem mass spectrometry technology, followed by data processing and stringent quality control procedures, ultimately retaining metabolites with ≥50% availability across the cohort.^[21]^

The resting-state functional magnetic resonance imaging data in this research were sourced from 4 cohort studies: UK Biobank, Adolescent Brain Cognitive Development, Philadelphia Neurodevelopmental Cohort, and Human Connectome Project. A total of 47,276 individuals were included in these studies. The data consisted of resting-state functional magnetic resonance imaging images, which were processed following a unified pipeline to generate 76 amplitude traits reflecting regional neuronal activity and 1695 pairwise functional connectivity traits quantifying interregional coactivity, alongside 6 global functional connectivity measures. Considering that genetic factors exert less influence on brain functional networks compared with structural aspects, the researchers conducted a GWAS on 1777 neuroimaging phenotypes to discover genetic variants influencing intrinsic brain activity. Consequently, 191 traits were identified as significantly impacted by genetic variations, comprising 75 amplitude traits (nodes), 111 pairwise functional connectivities (edges), and 5 global functional connectivities. These phenotypes covered various networks, including salience, default mode, central executive, somatomotor, attention, limbic, and visual networks.^[22]^

The GWAS statistics for allergic asthma (AA), AR, allergic purpura (AP), allergic conjunctivitis (AC), allergic contact dermatitis (ACD), allergic urticaria (AU), and pollen allergy (PA) were obtained from data released by FinnGen Research (https://r12.finngen.fi/) on November 4 2024, the diagnostic criteria for AA were based on International Classification of Diseases, 10th revision (ICD-10) standards, and the GWAS statistics for AR contain 16,383,313 loci of variation from 8430 cases and 298,829 controls. The diagnostic criteria for AR were based on ICD-9 and ICD-10 standards, and the GWAS statistics for AR contain 16,383,313 loci of variation from 8430 cases and 298,829 controls. The diagnostic criteria for AP were based on ICD-10 standards, and the GWAS statistics for AP contain 493,415 loci of variation from 1140 cases and 492,275 controls. The diagnostic criteria for AC were based on ICD-10 standards, and the GWAS statistics for AC contain 500,348 loci of variation from 29,791 cases and 470,557 controls. The diagnostic criteria for ACD were based on ICD-10 standards, and the GWAS statistics for ACD contain 438,800 loci of variation from 5926 cases and 432,874 controls. The diagnostic criteria for AU were based on ICD-10 standards, and the GWAS statistics for AU contain 486,216 loci of variation from 3315 cases and 482,901 controls. The diagnostic criteria for PA were based on ICD-10 standards, and the GWAS statistics for PA contain 496,215 loci of variation from 8776 cases and 487,439 controls (Table 1).

**Table 1:** Sample characteristics and data sources of phenotypes.

Phenotype	Sample size	Ancestry	ID
Gut microbiota abundance	7738	European	GCST90032172-GCST90032644
Cerebrospinal fluid metabolites	689	European	GCST90025999-GCST90026336
Brain networks	47,276	European	NA
Allergic asthma	283,740	European	finngen_R12_ALLERG_ASTHMA
Allergic rhinitis	490,219	European	finngen_R12_ALLERG_RHINITIS
Allergic purpura	493,415	European	finngen_R12_D3_ALLERGPURPURA
Allergic conjunctivitis	500,348	European	finngen_R12_H7_ALLERGICCONJUNCTIVITIS
Allergic contact dermatitis	438,800	European	finngen_R12_L12_ALLERGICCONTACT
Allergic urticaria	486,216	European	finngen_R12_L12_URTICA_ALLERG
Pollen allergy	496,215	European	finngen_R12_POLLENALLERG

The ID of “brain networks” is marked as “NA” because the original GWAS study on brain functional networks (Zhao et al^22^, Nat Genet) only provided summary statistics and did not publicly release specific phenotype IDs.

Supplementary data sources: Gut microbiota data were retrieved from the EBI GWAS Database (https://www.ebi.ac.uk/gwas/) with phenotype IDs ranging from GCST90032172 to GCST90032644; the sample size of 7738 represents valid analytical samples (excluding samples with missing microbiota taxonomic annotations); GWAS statistics for allergic diseases were obtained from the FinnGen Research R12 database (https://r12.finngen.fi/, released on November 4, 2024). All disease diagnoses adhered to ICD-9/10 criteria, and all samples were of European ancestry to avoid population stratification bias.

GWAS = genome-wide association studies.

To address the limited number of genome-wide significant SNPs (*P* < 5 × 10^−8^) available for GM and CSF metabolite GWASs, we adopted a dual-threshold strategy, selecting SNPs at *P* < 5 × 10^−6^ for GM and *P* < 1 × 10^−5^ for CSF metabolites.^[23]^ This approach is commonly used in microbiome- and metabolome-based MR studies, where genetic effects are typically modest and sample sizes relatively small. Using less stringent thresholds helps retain an adequate number of instruments and prevents a substantial loss of statistical power.

**Figure 2.**
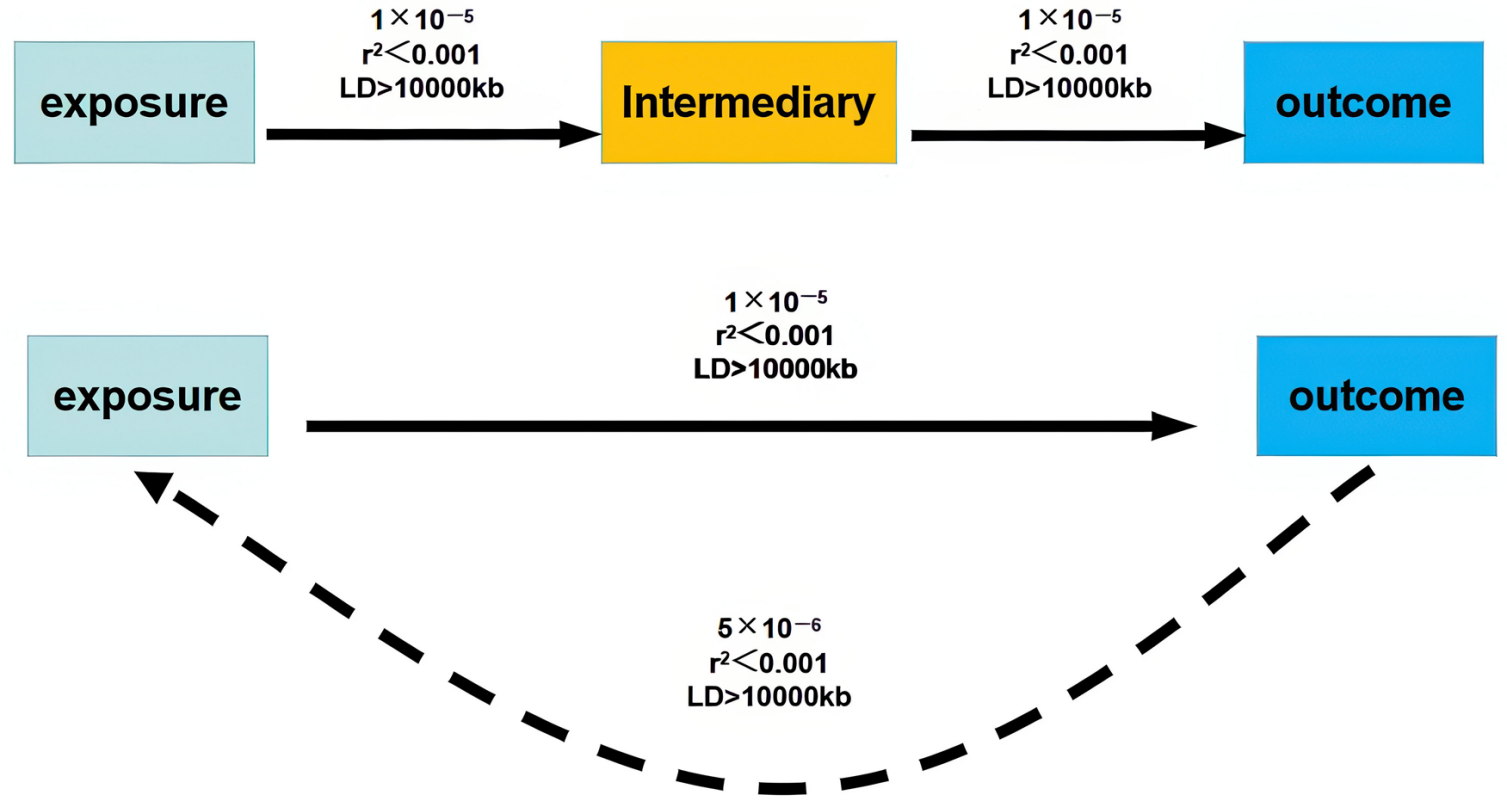
Depicts the Mendelian randomization causal pathways with single nucleotide polymorphism (SNP) selection criteria, including significance thresholds, linkage disequilibrium (LD) correlation (*r*^2^ < 0.001), and LD distance (>10,000 kb). LD = linkage disequilibrium, SNP = single nucleotide polymorphism.

To ensure robustness while minimizing the risk of false positives, several quality control procedures were implemented. First, linkage disequilibrium (LD) pruning was performed to meet the independence assumption of 2-sample MR, using *r*^2^ < 0.001 and a clumping window of >10,000 kb to remove correlated variants. Instrumental variables associated with known risk factors for ADs were further excluded using LDlink to reduce potential horizontal pleiotropy. Palindromic or incompatible SNPs were removed during harmonization, and exposure SNPs without corresponding outcome statistics were discarded (Fig. 2).

Second, instrumental strength was assessed using the F statistic (*F* = β^2^_exposure/standard error^2^_exposure),^[24–26]^ and SNPs with *F* < 10 were eliminated to avoid weak instrument bias.^[27]^

Third, multiple testing correction was performed using the false discovery rate (FDR), and only associations that remained significant after FDR adjustment were retained.

Finally, the robustness of the causal estimates was examined through several sensitivity analyses, including the MR-Egger intercept test, Cochran *Q* test, and MR-pleiotropy residual sum and outlier (PRESSO), none of which suggested substantial horizontal pleiotropy or heterogeneity. Collectively, these procedures justify the threshold selection and enhance the reliability of the causal inferences.

### 2.3. Statistical methods

All procedures were performed in R 4.4.2 (R Foundation for Statistical Computing, https://www.r-project.org). The 2-sample MR analysis was performed, and the results were visualized using the “Two Sample MR” (version 0.6.8; MRC Integrative Epidemiology Unit, University of Bristol) package, the “ggplot2” package (Posit/RStudio) and “ieugwasr.” Our MR study obtained significant SNPs associated with CSF metabolites, GM and ADs from publicly available GWAS. These SNPs meet the above 3 assumptions at the same time. LDlink helped to retrieve the above obtained SNPs.

### 2.4. Sensitivity analysis

We combined SNP estimates using IVW meta-analysis. IVW improves the precision of the estimates and the testing power. We performed horizontal pleiotropy tests using MR-Egger, which is the most classical MR analysis that identifies horizontal pleiotropy because of the consideration of intercepts. Cochran *Q* was used to assess heterogeneity among individual genetic variants. We also performed weighted median and outlier (PRESSO) analyses. The MR-PRESSO global test identifies outliers, and when *P* < .05, it indicates the presence of outliers, which should be excluded, and the MR analysis should be performed again until *P* > .05. Finally, “leave-one-out analysis” was used to further explore the effect of other genetic variations on the results (Mendel randomly included several SNPs of instrumental variables, and by excluding SNPs one by one and observing whether the effect values changed significantly, we could test whether the results were affected by a single SNP, thus reflecting the robustness of the conclusions). The “product of co-efficients” method was used to assess the indirect effect of GM on AR risk through potential mediators. The standard error of the indirect effect was determined using the delta method.

### 2.5. Selection of genetic instrumental variables

On the basis of previous studies and because of the limited number of SNPs screened, it is appropriate to set the significance threshold of instrumental variables (IVs) for each CSF metabolite trait to 1 × 10^−5^, and for GM to 5 × 10^−6^, ensuring that the selected IVs are completely independent of any potential confounders. We clustered SNPs by removing chain imbalances (LD, *R*^2^ < 0.001 and within 1000 kb); after this criterion, SNPs were screened to ensure the independence of the IVs. The above criteria were repeatedly validated in previous studies. To eliminate errors because of weak instrumentation, we calculated the *R^2^* and *F* statistics for each SNP with an intercept of 10, and excluded those SNPs with weak associations with *F* < 10 to ensure the robustness of the results and the screening of assessment IVs (*F* > 10 is considered sufficiently informative for MR analyses). Notably, none of the SNPs we used overlapped, and no proxy SNPs were used. With the above sensitivity analyses, we removed SNPs that were potentially confounders associated with GM and AR. The final results suggested that the *F*-statistics for IVs ranged from 19 to 30.65, evidence of no weak instrumental variables or the presence of IVs bias. Detailed information about IVs is provided in Tables S4 and S5, Supplemental Digital Content 3 and 4.

To improve the accuracy of hypothesis testing and to avoid false positives, we corrected the *P* values for FDR. The FDR-corrected *P* value was <.05, which further supports the strong robustness of our conclusions.

## 3. Results

### 3.1. Total effect of GM on AR

**Figure 3.**
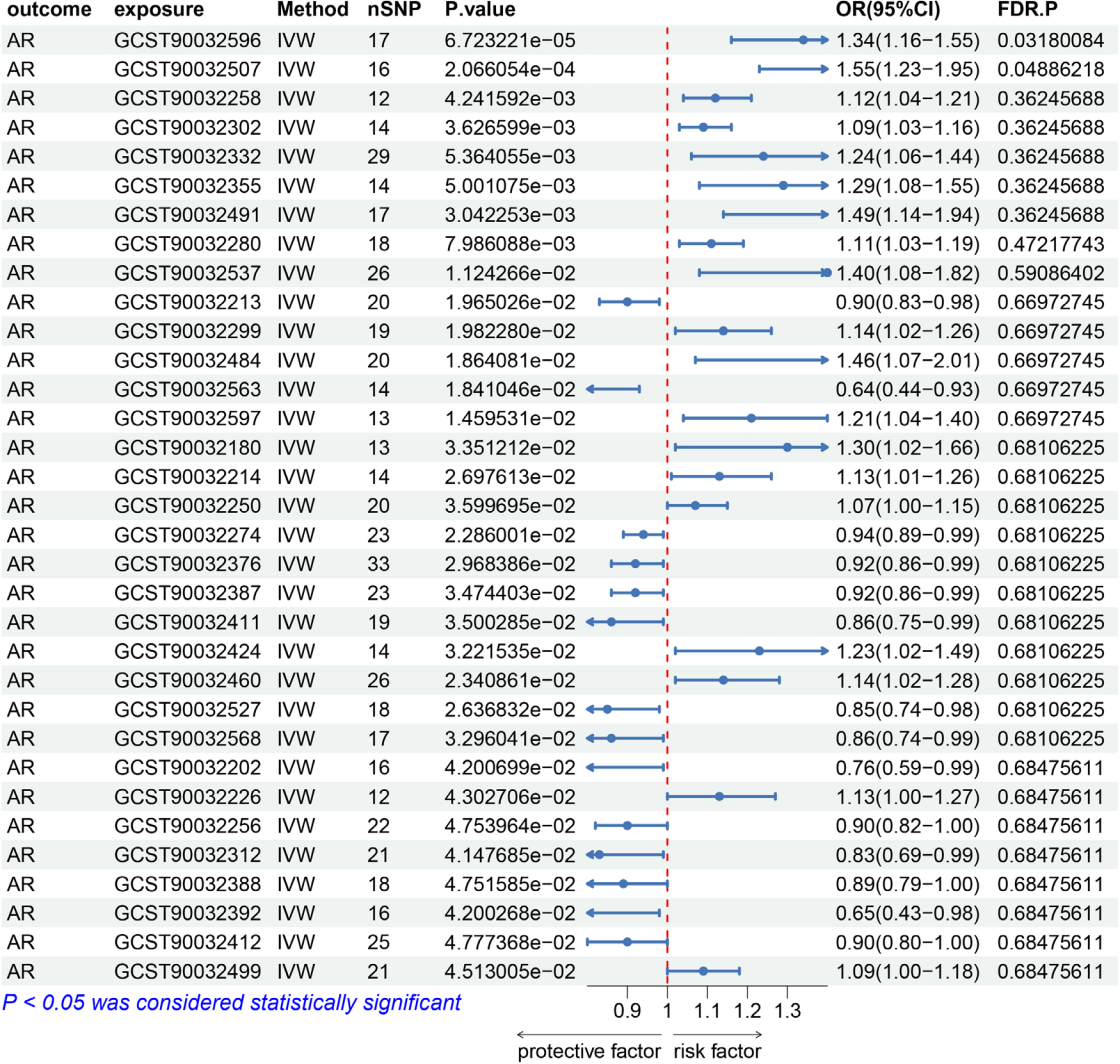
Forest plot of genetic causality between CSF metabolites and AR. AR = allergic rhinitis, CSF = cerebrospinal fluid, CI = confidence interval, FDR = false discovery rate, IVW = inverse variance weighted, OR = odds ratio, SNPs = single nucleotide polymorphisms.

To evaluate the overall causal effect of GM on AR, we performed MR analyses using 5 complementary methods, with the IVW approach as the primary estimator. After controlling for multiple testing using FDR correction, only 2 microbial features, UBA11963 sp002362595 and *Peptococcia*, remained significantly associated with AR (FDR-adjusted *P* < .05). A total of 473 gut microbial features were initially examined, of which 33 reached nominal significance (*P* < .05) for AR, but only the 2 aforementioned features survived FDR correction (Fig. 3).

Preliminary MR analyses using IVW indicated positive causal effects of these exposures on AR. Specifically, increased stool abundance of UBA11963 sp002362595 was associated with higher AR risk (odds ratio [OR] = 1.34; 95% confidence interval [CI]: 1.16–1.55; *P* = 6.72 × 10^−5^; FDR *P* = .0318), and increased *Peptococcia* abundance was also associated with elevated risk (OR = 1.55; 95% CI: 1.23–1.95; *P* = 2.0 × 10^−4^; FDR *P* = .0489). Both the MR-Egger intercept and Cochran *Q* tests indicated no significant horizontal pleiotropy or heterogeneity (all *P* > .05), supporting the robustness of these findings. The Steiger directionality test confirmed the correct causal direction for both exposures (*P* < .001). Results were consistent across complementary sensitivity analyses, reinforcing the validity of the observed causal effects.

These findings suggest that genetically driven increases in specific gut microbial abundances may constitute potential risk factors for AR, providing genetic-level evidence for exploring AR pathogenesis via the microbiota-gut-brain axis. Consequently, UBA11963 sp002362595 and *Peptococcia* were selected as primary exposures for subsequent mediation analyses to investigate whether their effects on AR are mediated through cerebrospinal fluid metabolites. The detailed MR results are presented in Tables S1–S3, Supplemental Digital Content 1.

### 3.2. Reverse MR analysis

**Figure 4.**
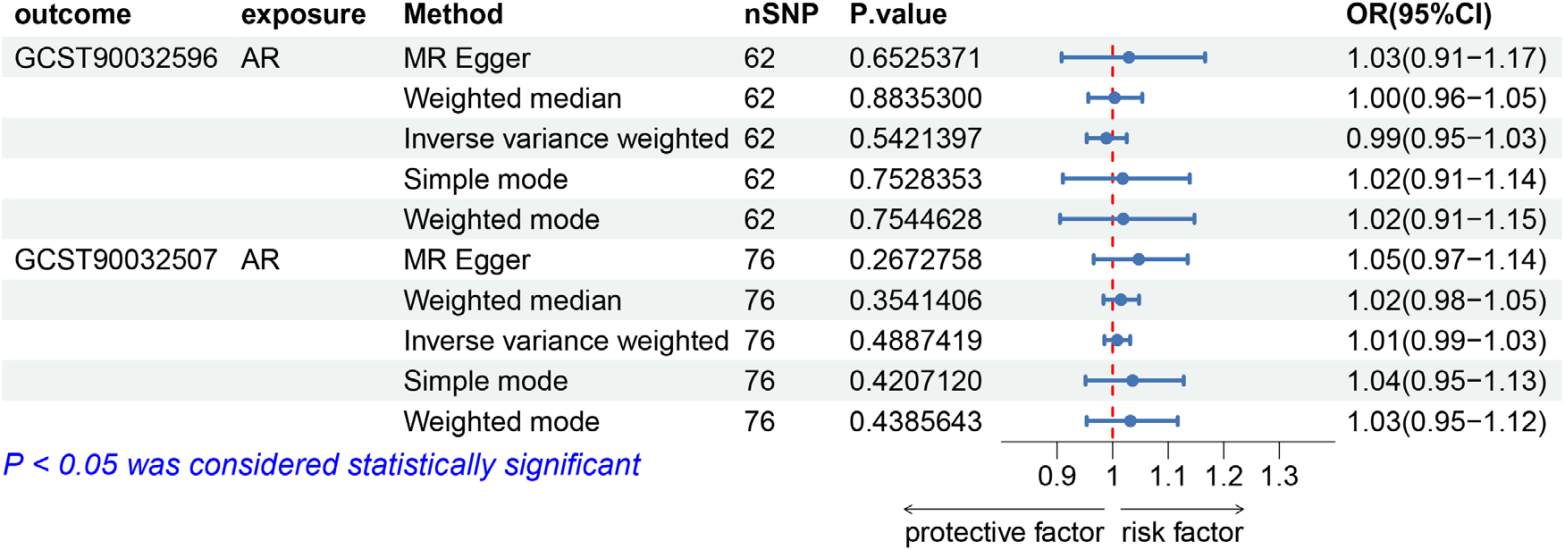
Forest plot of the results of inverse MR analysis of genetic causality between GM and AR. AR = allergic rhinitis, CI = confidence interval, GM = gut microbiota, MR = Mendelian randomization, OR = odds ratio, SNPs = single nucleotide polymorphisms.

We found a potential causal role for UBA11963 sp002362595 and *Peptococcia* abundance in stool in AR. Next, we performed reverse MR analysis to assess the causal effect of AR on UBA11963 sp002362595 and *Peptococcia* abundance in stool. The results showed no significant causal effect (*Peptococcia* inverse variance weighted method: OR = 1.014, 95% CI = 0.985–1.032, *P* = .489; UBA11963 sp002362595 inverse variance weighted method: OR = 0.989, 95% CI = 0.953–1.025, *P* = .542). MR-Egger intercept test and Cochran *Q* test did not reveal any evidence of horizontal pleiotropy or heterogeneity (all *P* values > .05). In addition, no outliers were detected in sensitivity analyses. All *P* values were >.05, indicating that the results were not statistically significant (Fig. 4). The detailed SNP are presented in Table S7, Supplemental Digital Content 2.

### 3.3. GM effects on CSF metabolites

**Figure 5.**
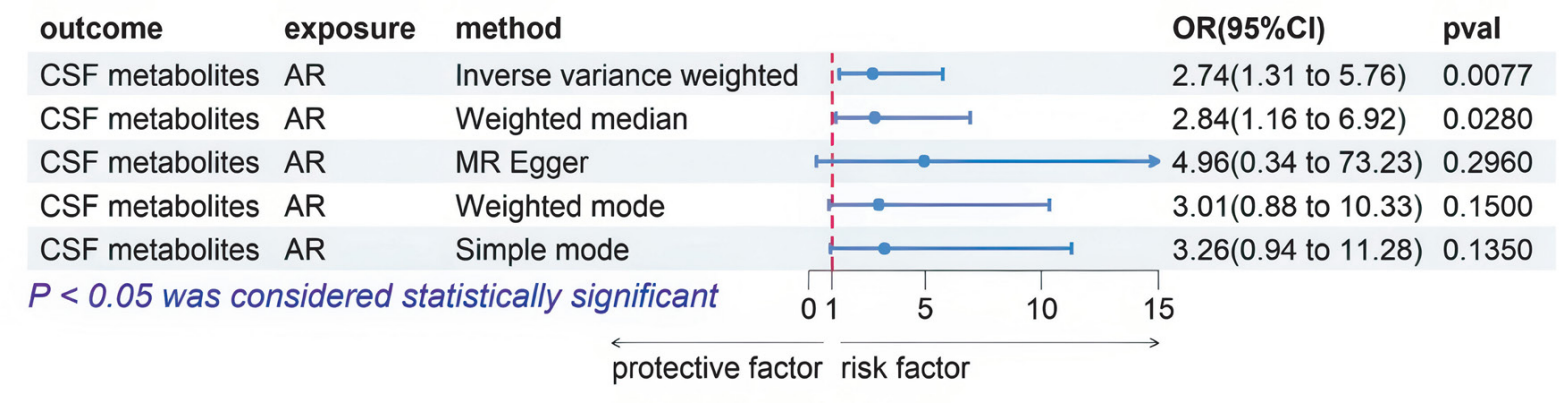
Forest plot of genetic causality between GM and CSF metabolites. CI: confidence interval, GM = gut microbiota, MR = Mendelian randomization, OR = odds ratio; SNPs = single nucleotide polymorphisms.

Our previous studies have shown that 2 groups of GM have a significant influence on AR. We further explored the role of these GM on CSF metabolites. The IVW MR results showed that the abundance of Peptococcaceae only in stool was significantly associated with CSF metabolite methyl succinoyl-carnitine levels (OR = 2.74, 95% CI = 1.31–5.76, *P* = .0077, pFDR < .05). A similar significant causal relationship was also observed using the weighted median method (OR = 2.84, 95% CI = 1.16–6.92, *P* = .028). However, no significant associations were found with other MR methods (MR-Egger: OR = 4.96, 95%CI = 0.34–73.23, *P* = .296; weighted mode: OR = 3.01, 95% CI = 0.88–10.33, *P* = .150; simple mode: OR = 3.26, 95% CI = 0.94–11.28, *P* = .135). Importantly, no evidence of horizontal pleiotropy was detected (MR-Egger intercept *P* = .672), and heterogeneity tests showed consistent results (Cochran *Q P* > .98), indicating the robustness of our findings (Fig. 5). The detailed SNP are presented in Table S4, Supplemental Digital Content 3.

### 3.4. Effect of CSF metabolite characterization on AR

We used MR to evaluate the causal effects of CSF metabolites, Methyl succinoyl-carnitine levels, on AR. The IVW method revealed significant positive causal effects for metabolites: Methyl succinoyl-carnitine levels increased the risk of AR (OR = 1.04, 95% CI = 1.02–1.07, *P* = 1.11 × 10^−5^, pFDR < .05). For Methyl succinoyl-carnitine levels, significant associations were further supported by the weighted median method (OR = 1.05, 95% CI = 1.02–1.08, *P* = .000529) and MR-Egger method (OR = 1.05, 95% CI = 1.01–1.09, *P* = .022). Importantly, the MR-Egger intercept test and Cochran *Q* test indicated no evidence of horizontal pleiotropy or heterogeneity (all *P* values > .05), and sensitivity analyses confirmed the robustness of the findings.

**Figure 6.**
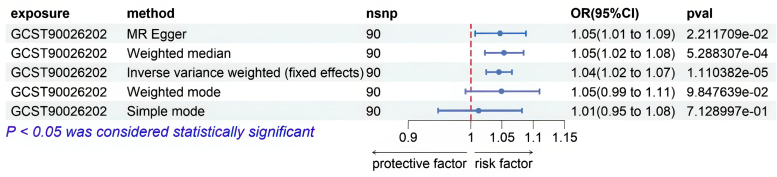
Forest plot of genetic causality between CSF metabolites and AR. AR = allergic rhinitis, CSF = cerebrospinal fluid, CI = confidence interval, MR = Mendelian randomization, OR = odds ratio, SNPs = single nucleotide polymorphisms.

Using MR, we found that genetically elevated CSF metabolite Methyl succinoyl-carnitine levels exerted a significant positive causal effect on the risk of AR. The primary IVW analysis indicated that each standard deviation increase in genetically predicted methyl succinoyl-carnitine was associated with a 4% higher AR risk (OR = 1.04, 95% CI = 1.02–1.07, *P* = 1.11 × 10^−5^, FDR < 0.05). This causal association was consistently supported by complementary methods: the weighted median method (OR = 1.05, 95% CI = 1.02–1.08, *P* = 5.29 × 10^−4^) and the MR-Egger method (OR = 1.05, 95% CI = 1.01–1.09, *P* = .022). Importantly, sensitivity analyses revealed no evidence of horizontal pleiotropy (MR-Egger intercept *P* > .05) or heterogeneity (Cochran *Q P* > .05), confirming the robustness of these findings (Fig. 6). The detailed results are presented in Tables S5 and S6, Supplemental Digital Content 4.

### 3.5. CSF metabolites mediate the mediation of GM on AR

In the genetic analysis, we analyzed the causal effects of GM on AR and CSF metabolites on AR. After identifying the significant mediators affecting AR and the subsequent effects of exposure on the mediators, we performed a mediator MR analysis to quantify the proportion of mediator effects to characterize the mediating role of CSF metabolites on GM on AR. The mediating role of CSF metabolites in the causal pathway from GM to AR was 9.02%. As can be seen, a statistically significant indirect effect (*P* = .040) was observed, indicating a partial mediation. In our results, the total effect *c* (OR = 1.55, 95% CI = 1.23–1.95), the indirect effect *a* (OR = 2.74, 95% CI = 1.31–5.76), and the indirect effect *b* (OR = 1.008, 95% CI = 1.02–1.07) were all significant, and thus the direct effect *c*’ should also be significant. The direct effect (*c*’ = 0.3985) was same in sign to the mediating effect (*ab* = 0.0395), and |*c*| > |*c*’| (0.438 > 0.3985), so there was no masking effect between GM and AR (Table 2).

**Table 2:** Mediation analysis results for methyl succinoyl-carnitine levels in abundance effect on AR.

Effect	Pathway definition	β	95% CI	*P* value
Total effect, *c*	*Peptococcia* → AR	0.440	0.210–0.670	2.07 × 10^−4^
*a* path	*Peptococcia* → methyl succinoyl-carnitine	1.008	0.270–1.751	7.70 × 10^−3^
*b* path	Methyl succinoyl-carnitine → AR	0.040	0.020–0.068	1.11 × 10^−5^
Indirect effect, *ab*	*Peptococcia* → methyl succinoyl-carnitine → AR	0.040	0.003–0.083	.040
Direct effect, *c′*	*Peptococcia* → AR, excluding methyl succinoyl-carnitine	0.400	0.170–0.630	6.53 × 10^−4^
Mediated proportion	*ab*/ *c* × 100%	9.02%		

**Effect definitions:** The *total effect (c*) represents the overall causal effect of *Peptococcia* abundance on AR. The *a* path represents the causal effect of *Peptococcia* on CSF methyl succinoyl-carnitine levels. The *b* path represents the causal effect of methyl succinoyl-carnitine on AR. The *indirect effect (ab*) represents the mediated causal effect of *Peptococcia* on AR through methyl succinoyl-carnitine, estimated as β(*a*) × β(*b*). The *direct effect (c′*) represents the residual causal effect of *Peptococcia* on AR independent of the mediator, estimated as β(*c*) − β(*ab*).

**Statistical methods:** All β values and 95% CIs were derived from inverse-variance weighted (IVW) 2-sample Mendelian randomization. The indirect effect (*ab*) and its 95% CI were estimated using the product-of-coefficients method with standard errors calculated via the delta method. The *P* value for the indirect effect was derived accordingly. The mediated proportion (9.02%) was calculated using unrounded β values as [β(*ab*)/ β(*c*)] × 100%. OR equivalents can be obtained as OR = e^β^; for example, β(*c*) = 0.440 corresponds to OR = 1.55 (95% CI: 1.23–1.95).

**Interpretation note:** The lower bound of the indirect effect CI (0.003) approaches zero, indicating that this mediation estimate is statistically marginal. These findings should be interpreted with caution and considered preliminary pending replication and experimental validation.

AR = allergic rhinitis, CI = confidence interval, CSF = cerebrospinal fluid, OR = odds ratio, β = regression coefficient (log-odds ratio scale).

### 3.6. Extended analysis

To investigate whether the onset of ADs is influenced by the brain, we conducted further MR analyses to explore the associations of brain functional networks and cerebrospinal fluid metabolites with 7 ADs (AA, AR, AP, AC, ACD, AU, PA). After sensitivity analysis and FDR correction, the following results were obtained:

Two-sample MR analyses using various brain-related omics datasets and ADs revealed that in the CSF metabolites-ADs analysis, several diseases exhibited nominally significant causal relationships (*P* < .05) in the initial analysis. However, after FDR correction, only partial associations remained statistically significant. Specifically, in the analysis of CSF metabolomics and ADs, 2 metabolites showed positive associations with AR: methyl succinoyl-carnitine levels (OR = 1.04, 95% CI = 1.02–1.07, *P* = 1.11 × 10^−5^, pFDR = 0.0038) and 1-oleoyl-glycerophosphorylcholine (18:1) levels (OR = 1.15, 95% CI = 1.07–1.23, *P* = 2.63 × 10^−4^, pFDR = 0.0445); one metabolite (indoleacetate levels, OR = 1.09, 95% CI = 1.04–1.14, *P* = 1.11 × 10^−4^, pFDR = 0.0376) demonstrated a positive association with ACD; while methyl succinoyl-carnitine levels (OR = 1.06, 95% CI = 1.03–1.09, *P* = 1.04 × 10^−5^, pFDR = 0.0035) were positively associated with PA, with all reported FDR values remaining statistically significant (pFDR < 0.05). No significant metabolites were identified for other ADs after FDR correction.

**Figure 7.**
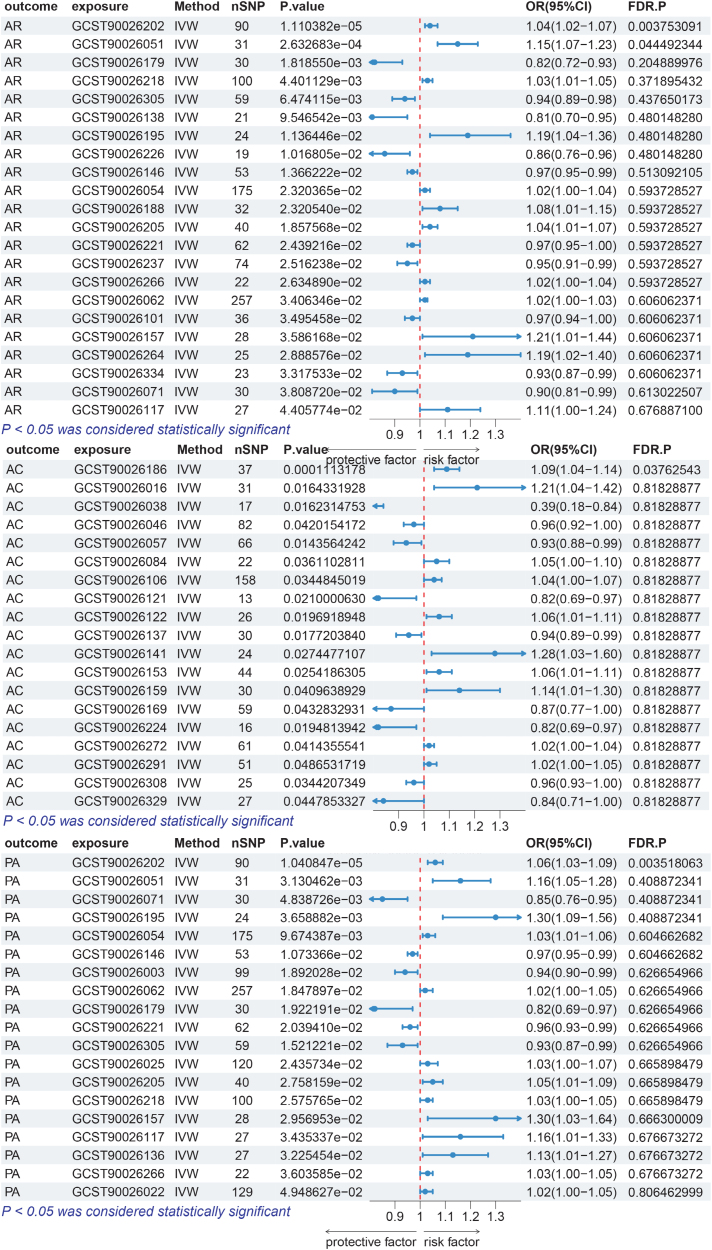
Forest plot of genetic causality between CSF metabolites and ADs. AR = allergic rhinitis, AC = allergic conjunctivitis, ADs = allergic diseases, CI = confidence interval, CSF = cerebrospinal fluid, FDR = false discovery rate, IVW = inverse-variance weighted, OR = odds ratio, PA = pollen allergy, SNPs = single nucleotide polymorphisms.

**Figure 8.**
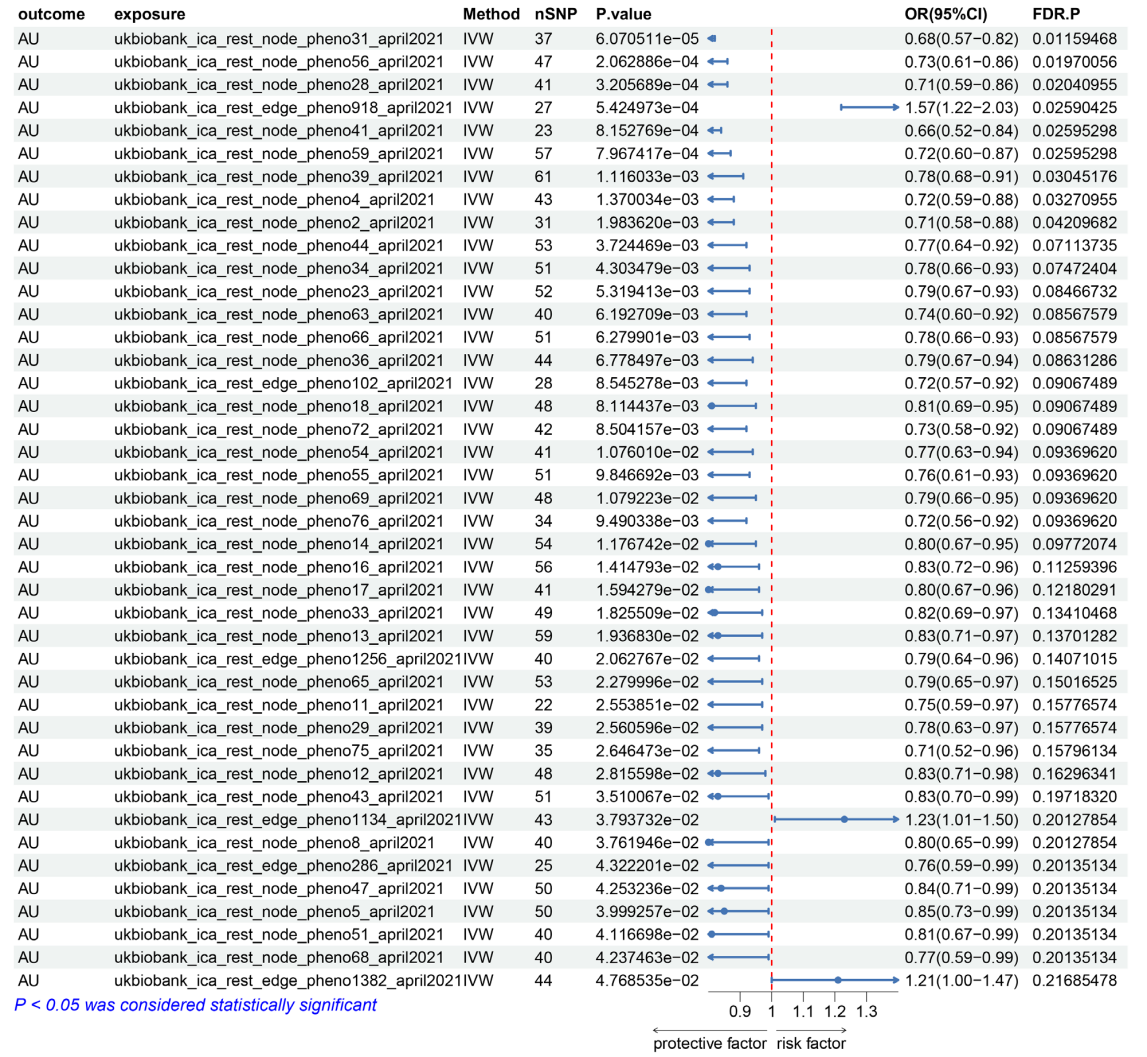
Forest plot of genetic causality between CSF metabolites and ADs. CI = confidence interval, FDR = false discovery rate, IVW = inverse-variance weighted, OR = odds ratio, SNPs = single nucleotide polymorphisms,

In the brain functional network analysis, only AU demonstrated significant negative associations after FDR correction, involving 9 functional networks: Occipital|Precuneus (OR = 0.68, 95% CI = 0.57–0.82, *P* = 6.07 × 10^−5^, pFDR = .0116), Postcentral|Precentral (OR = 0.73, 95% CI = 0.61–0.86, *P* = 2.06 × 10^−4^, pFDR = .0197), Frontal|Cingulate (OR = 0.71, 95% CI = 0.59–0.86, *P* = 3.21 × 10^−4^, pFDR = .0204), Precuneus|Angular|Cingulate (OR = 1.57, 95% CI = 1.22–2.03, *P* = 5.42 × 10^−4^, pFDR = .0259), Precuneus|Parietal_Sup (OR = 0.66, 95% CI = 0.52–0.84, *P* = 8.15 × 10^−4^, pFDR = .0260), Subcortical (OR = 0.72, 95% CI = 0.60–0.87, *P* = 7.97 × 10^−4^, pFDR = .0260), Postcentral|Precentral (OR = 0.78, 95% CI = 0.68–0.91, *P* = 1.12 × 10^−3^, pFDR = .0305), Parietal (OR = 0.72, 95% CI = 0.59–0.88, *P* = 1.37 × 10^−3^, pFDR = 0.0327), and Calcarine|Lingual|Cuneus (OR = 0.71, 95% CI = 0.58–0.88, *P* = 1.98 × 10^−3^, pFDR = .0421). No FDR-corrected significant results were observed for other ADs in the functional network analysis (Figs. 7 and 8). The detailed MR results are presented in Tables S8 and S9, Supplemental Digital Content 5.

## 4. Discussion

In recent years, substantial evidence has indicated that GM dysbiosis extensively influences immune diseases and neurological functions through the “gut-brain axis.”^[28]^ The communication mechanisms of this axis are complex, involving multiple pathways such as the vagus nerve, immune cell migration, and humoral diffusion of microbial metabolites.^[29]^ Among these, metabolites capable of freely crossing the blood–brain barrier or modulating its permeability are considered key messengers in remote regulatory processes, as they can directly act on the CNS.^[30]^ The pathogenesis of ADs is also closely associated with such neuroimmune crosstalk.^[31]^ In particular, Th2-type immune responses and neuropeptide-mediated inflammatory pathways in AR have been preliminarily identified.^[32]^ However, whether GM regulates AR through central humoral factors, such as cerebrospinal fluid metabolites, remains largely unexplored. Although previous studies suggest that GM may influence ADs by modulating immune balance, the specific causal pathways and the role of central mediators remain unclear.^[33,34]^ There is a particular lack of systematic validation regarding CSF metabolites as mediators between GM and AR.

This study systematically evaluates the causal effect of GM on AR and quantifies the mediating role of CSF metabolites in this pathway using 2-sample MR and mediation analysis, thereby addressing a critical gap in the “gut-brain-immune” triad mechanism. Subsequent extended analyses explored the potential effects of brain functional networks and brain volume on 7 ADs.

Our MR analyses, primarily using the inverse-variance weighted method, identified significant causal associations between 2 GM taxa (UBA11963 sp002362595 in feces and *Peptococcia* abundance) and AR. Reverse MR analyses did not indicate causal effects of AR on GM, supporting the reliability of the inferred causal direction. Further mediation analysis revealed that *Peptococcia* influences AR by regulating methyl succinyl-carnitine levels in CSF, with a mediation proportion of 9.02%. It should be noted, however, that the lower bound of the 95% CI for the indirect effect was 0.003, close to zero, and the *P* value was borderline (*P* = .040), indicating statistical fragility of this estimate. This mediation proportion should therefore be interpreted with caution and regarded as preliminary genetic evidence rather than a confirmed biological pathway. This finding provides genetic evidence consistent with a partial mediating role of CSF metabolites in the GM–AR pathway, offering a hypothesis for further investigation into the function of central humoral factors in ADs.

On a mechanistic level, we propose a hypothesis: *Peptococcia*, as anaerobic Gram-positive bacteria,^[35]^ may – through gut dysbiosis – alter host bile acid metabolism or dietary fiber fermentation, indirectly affecting mitochondrial fatty acid β-oxidationefficiency. This could lead to the accumulation of intermediate metabolites such as methyl succinyl-carnitine in CSF. Direct experimental evidence supporting methyl succinoyl-carnitine as an active signaling molecule rather than a passive metabolic intermediate is currently absent, and its putative effects on mast cell activation and neurogenic inflammation remain inferential at this stage. Nonetheless, these associations, identified through genetic proxies, provide a biologically plausible framework that may guide targeted mechanistic investigation. This hypothesis aligns with the previously proposed “metabolism-immune axis” theory, suggesting that GM-derived metabolites can remotely regulate peripheral immune responses via humoral pathways.^[36]^ More importantly, it bridges classical metabolic disorder theory with neuroimmunology, suggesting a potential pathological axis linking GM, central metabolism, and peripheral immunity that warrants further validation.

Furthermore, our results are consistent with multiple neuroimmune studies. For instance, clinical observations have reported abnormal levels of inflammation-related metabolites in the CSF of AR patients.^[37]^ Another animal study confirmed that GM modulation can alter central metabolic profiles and alleviate allergic symptoms.^[38]^ These consistencies enhance the credibility of our findings, suggesting that the GM–CSF metabolite pathway may be a common mechanism in AR pathogenesis.

In extended analyses, CSF metabolites showed positive associations with AR, allergic contact dermatitis, and pollen allergy, whereas brain functional networks were significantly negatively correlated only with allergic urticaria (involving regions such as the occipital lobe and anterior cingulate cortex). This implies that sensory integration and autonomic regulation may participate in the pathological process of urticaria. The lack of significant associations with brain volume may reflect weaker effects of structural brain changes on ADs, potentially requiring longer-term cumulative influences. These findings broaden the scope of the “gut-brain-immune” axis and provide preliminary clues to the neuroregulatory mechanisms in specific ADs such as urticaria.

The significance of this study lies in offering new targets for the precise prevention and treatment of AR. For example, dietary interventions to modulate *Peptococcia* abundance or interventions targeting methyl succinyl-carnitine metabolism may serve as auxiliary therapies, consistent with research on fecal microbiota transplantation for AR improvement.^[39]^ In addition, the association between brain functional networks and allergic urticaria suggests that behavioral therapies such as biofeedback training could be applicable to ADs.^[40]^

In a broader scientific context, although decades of research have advanced the understanding of AR’s biological basis, most findings rely on animal models, and their applicability to humans remains debated because of inherent limitations. By leveraging genetic instruments in human population data, our study overcomes this constraint. The results highlight the significant impact of GM on CSF metabolites, reinforce the potential therapeutic relevance of their interaction, and provide new directions for diagnostics (e.g., laboratory biomarkers), treatments, or secondary prevention based on metabolite-exposure correlations.

These findings not only open new avenues for investigating the roles of GM and CSF metabolites in AR but also demonstrate practical applicability: for patients, our results may aid self-regulation and prevention; for clinicians, CSF metabolites represent candidate markers worthy of investigation in neuroimmune subtypes of AR, pending replication; for educators, this study offers valuable reference material; and for researchers, the elucidated GM–CSF metabolite–AR pathway lays the foundation for developing “microbiota-metabolism” bidirectional regulatory drugs.^[41]^

This study provides genetic evidence that the causal effect of GM on AR is partially mediated through CSF metabolites and reveals the regulatory role of brain functional networks in ADs. These findings not only deepen the understanding of the “gut-brain-immune” axis but also complement existing research, offering new directions for stratified diagnosis and immune interventions in ADs.

## 5. Conclusion

This MR study provides genetic evidence consistent with a potential causal pathway linking GM, CSF metabolites, and AR. Specifically, genetically predicted higher *Peptococcia* abundance was associated with increased AR risk, with a modest proportion (9.02%) of this effect potentially mediated through CSF methyl succinoyl-carnitine levels; however, this mediation estimate should be interpreted cautiously given its statistical marginality. Extended analyses further suggest that brain functional networks may participate in the pathogenesis of certain ADs. These findings offer a preliminary framework for the “gut-brain-immune” axis in AR and warrant replication and experimental validation.

## 6. Limitations

This study has several limitations. First, the use of relaxed instrument selection thresholds (*P* < 1 × 10^−5^) for certain exposures may increase the risk of weak instrument bias and false-positive associations, and findings derived from such analyses require independent replication. Second, although sensitivity analyses did not indicate significant directional pleiotropy, the wide confidence intervals observed in MR-Egger estimates reflect limited statistical precision, and residual horizontal pleiotropy cannot be fully excluded. Finally, as a genetics-only study, this work cannot validate biological mechanisms; the proposed gut–brain–immune pathway should be regarded as a speculative framework requiring experimental validation.

## Acknowledgments

We want to acknowledge the participants and investigators of the FinnGen study. The successful advancement of this research would not have been possible without the generous support and selfless contributions of all data providers, to whom we extend our most sincere gratitude.

## Author contributions

**Conceptualization:** Guoteng Zhao.

**Data curation:** Guoteng Zhao.

**Formal analysis:** Guoteng Zhao.

**Writing – original draft:** Guoteng Zhao.

**Funding acquisition:** Peizheng Yan, Zhenguo Wang.

**Investigation:** Peizheng Yan.

**Resources:** Peizheng Yan, Zhenguo Wang.

**Validation:** Ran Qiu, Zhihao Zhang.

**Visualization:** Ran Qiu, Zhihao Zhang.

**Methodology:** Mingzhe Zhao.

**Supervision:** Zhenguo Wang.

**Writing – review & editing:** Zhenguo Wang.


















